# Genome-wide association study reveals genomic regions associated with *Pinus pinaster* response to pinewood nematode

**DOI:** 10.3389/fpls.2026.1765158

**Published:** 2026-03-13

**Authors:** Vera Inácio, Inês Modesto, Elsa Gonçalves, Ana Vila-Verde, Ana Milhinhos, José Antonio Cabezas, María Teresa Cervera, Isabel Carrasquinho, Célia M. Miguel

**Affiliations:** 1 BioISI – Instituto de Biosistemas e Ciências Integrativas, Faculdade de Ciências, Universidade de Lisboa, Lisbon, Portugal; 2 Instituto de Tecnologia Química e Biológica António Xavier, Universidade Nova de Lisboa, Oeiras, Portugal; 3 LEAF—Linking Landscape, Environment, Agriculture and Food, Instituto Superior de Agronomia, Universidade de Lisboa, Lisbon, Portugal; 4 Instituto de Ciencias Forestales, Instituto Nacional de Investigación y Tecnología Agraria y Alimentaria, Consejo Superior de Investigaciones Científicas (CSIC) (ICIFOR-INIA, CSIC), Madrid, Spain; 5 Instituto Nacional de Investigação Agrária e Veterinária, Oeiras, Portugal

**Keywords:** *Bursaphelenchus xylophilus*, GWAS, maritime pine, molecular markers, pine wilt disease, single nucleotide polymorphism

## Abstract

**Introduction:**

Pine wilt disease (PWD), caused by the pinewood nematode (PWN) *Bursaphelenchus xylophilus*, is a major threat to conifer forests worldwide and severely impacts *P. pinaster* Ait in the Iberian Peninsula. Although this species is highly susceptible to PWD, previous studies revealed genetic variability in response to PWN, suggesting potential for breeding programs.

**Methods:**

In this study, we assessed the susceptibility of five half-sib families and performed a genome-wide association study (GWAS) using a linear mixed model to identify genomic regions associated with *P. pinaster* response to PWN. A panel of 510 plants was inoculated under controlled conditions, and disease progression was quantified using the area under the disease progress curve (AUDPC). Single-nucleotide polymorphisms (SNPs) were genotyped in the 510-panel using a customized SNP array.

**Results:**

Significant family differences in susceptibility were detected, and six SNPs were associated with AUDPC, which mapped to genes involved in chloroplast function, immune signaling, and stress response. Notably, the genetic architecture of the response included both epistatic interactions between two *loci* and overdominance in susceptibility at two additional loci, highlighting the significant role of non-additive genetic effects.

**Discussion:**

These findings provide insights into the polygenic architecture of PWN response and identify candidate markers that, after validation, could support marker-assisted selection for maritime pine breeding programs.

## 1 Introduction

Pine wilt disease (PWD) is a highly impactful disease, typically leading to the death of affected pine trees within a few weeks to a few months. The causal pathogen is *Bursaphelenchus xylophilus*, commonly known as the pinewood nematode (PWN), and it is transmitted by the insect vector *Monochamus* spp. during its feeding activity on healthy trees. The feeding wounds provide entry portals for the PWN, which, once inside the stem, feeds on plant cells surrounding the resin ducts, disrupting the water transport and repeating its propagative cycle. As the PWN population increases, the water transport is significantly impeded, ultimately resulting in the death of the tree (Futai, 2013).

This disease poses a growing threat to conifer forests on a global scale, particularly in Asia and Southeastern Europe, with severe economic losses for the forestry industry and a significant environmental impact (Webster and Mota, 2008). Moreover, high temperature and drought periods, expected to become more frequent in many parts of the globe, further promote PWD rapid spread (Calvão et al., 2019). The emergence of PWD in Europe was first identified in Portugal in 1999 and, despite the implementation of sanitary measures, has subsequently expanded into Spain (Mota et al., 1999) and an outbreak was very recently reported in France by the French Agency for Food, Environmental and Occupational Health & Safety) national reference laboratory (https://www.anses.fr/en/content/pinewood-nematode-threat-conifers). However, the PWD differentially affects pine species, with *P. massoniana*, *P. thunbergii*, *P. densiflora*, *P. sylvestris* and *P. pinaster* showing susceptibility and *P. taeda*, *P. halepensis*, and *P. pinea* tolerance or resistance (Mamiya, 1983; Guo et al., 2023).

To cope with the harmful effects of PWD, breeding programs have been developed in Japan for *P. thunbergii*, *P. densiflora*, and *P. massoniana* by propagating resistant tree varieties (Nose and Shiraishi, 2008). Given the substantial economic and ecological value of *P. pinaster* in Southwestern Europe - due to its utilization in paper, timber, and resin production, its role in soil protection, and significance as wildlife habitat (Webster and Mota, 2008; Vicente et al., 2012)) - and its high susceptibility to PWN, the first steps of a breeding program have also been initiated in Portugal and Spain for this species (Ribeiro et al., 2012; Carrasquinho et al., 2018; Menéndez-Gutiérrez et al., 2018). In Portugal, apparently healthy trees were identified from a heavily affected area have been established as the reference population for PWD resistance/tolerance (Ribeiro et al., 2012). Different levels of susceptibility were observed in half-sib families selected from the breeding populations, after PWN artificial inoculation (Carrasquinho et al., 2018; Menéndez-Gutiérrez et al., 2018). Genetic selection tests have detected genetic variability in the response to PWN with an heritability of family means between 0.37–0.59 (Carrasquinho et al., 2018; Menéndez-Gutiérrez et al., 2018), revealing the potential for an effective breeding program.

The development of molecular markers for marker-assisted selection aiming to early discriminate between resistant and susceptible plants would greatly benefit the progress of breeding programs (Naidoo et al., 2019). Using SNPs for the identification of molecular makers that could be used for marker-assisted selection offers advantages, given their abundance in the genome, stability, robustness, and widespread distribution throughout the genome (Neale and Savolainen, 2004; Pavy et al., 2008, 2013; Badenes et al., 2016). The impact of next generation sequencing (NGS) and high throughput genotyping platforms for SNP discovery is particularly relevant in trees as they contribute to expediting the long breeding process and enhancing its overall efficiency (Grattapaglia and Resende, 2011; Pavy et al., 2013; Badenes et al., 2016; Zas et al., 2017). Pine species have extremely large genomes with high proportions of repetitive DNA, which has historically delayed the availability of high-quality reference genome sequences (De La Torre et al., 2014). The availability of a reference genome allows comprehensive genome-wide variant discovery, precise marker localization, and assessment of linkage relationships, thereby strengthening downstream genetic analyses. Nevertheless, in pine species lacking complete reference genomes, including *P. pinaster*, *de novo* transcriptome approaches have been widely applied providing valuable resources for exploring functional traits in non-model species (Parchman et al., 2010; Canales et al., 2014; Visser et al., 2015; Wachowiak et al., 2015; Rodrigues et al., 2018; Usié et al., 2022; Manjarrez et al., 2024) and to gain insights into conifers responses to pests or diseases (Liu et al., 2013; Visser et al., 2018; Kovalchuk et al., 2019; Hernandez-Escribano et al., 2020; Trujillo-Moya et al., 2020). In the case of the response to PWN, this approach has uncovered mechanisms highlighting the involvement of phytohormone signalling, including the jasmonic acid (JA) defence pathway, secondary metabolism pathways, such as terpene and lignin biosynthesis, oxidative stress response genes, and resistance genes (reviewed in (Modesto et al., 2022c)). In *P. pinaster*, SNP arrays have been created derived from EST or from re-sequenced genes (Lepoittevin et al., 2010; Chancerel et al., 2011; Plomion et al., 2016) and used in various studies, including nucleotide diversity analysis (Plomion et al., 2014), detection of QTLs for photosynthesis and water use efficiency (de Miguel et al., 2014), association mapping for growth, wood and fire-related traits (Lepoittevin et al., 2012; Budde et al., 2014; Bartholomé et al., 2016), growth performance (Cabezas et al., 2015), environmental association (Jaramillo-Correa et al., 2015), and linkage map construction (Chancerel et al., 2011; Plomion et al., 2016). Recently, a customized SNP array (4TREE Axiom array) including approximately 50,000 highly confident SNPs from *P. pinea*, P*. pinaster*, *Populus* sp and *Fraxinus sp* was developed (Guilbaud et al., 2020).

Despite the potential of marker-assisted selection, research on the identification of molecular markers linked to the phenotypic response to PWD is still limited. So far, only one study has addressed this topic in *P. pinaster*, finding two significant SNPs in two genes; however, significance was not maintained after stringent correction for multiple testing (Modesto et al., 2022a). Importantly, a major quantitative trait locus (QTL) controlling PWN resistance (*PWD1*) was found in the most resistant variety of *P. thunbergii*, after PWN inoculation (Hirao et al., 2019, 2022), although the nature of disease resistance in tree-pathogen interactions is usually polygenic (Younessi-Hamzekhanlu and Gailing, 2022; Demirjian et al., 2023).

Single-marker models for genome-wide association studies (GWAS) often led to reduced statistical power and biased estimates when dealing with complex genetic architectures, where quantitative traits are controlled by multiple loci with small individual effects (Segura et al., 2012). Therefore, GWAS using complex models have been successfully applied to detect genes statistically associated with a variety of relevant traits in trees, including *P. halepensis* (Urrestarazu et al., 2017; da Silva Linge et al., 2021; Santini et al., 2021; Giordani et al., 2022; Tan and Ingvarsson, 2022), and crop species (Bonnafous et al., 2018; Cui et al., 2018; Hu et al., 2018; Delfini et al., 2021; Malik et al., 2021; Zhong et al., 2021; Dang et al., 2022; Enyew et al., 2022; Vikas et al., 2022; Delfan et al., 2023).

In this work, we aimed at identifying genomic regions associated with the *P. pinaster* response to PWN through GWAS using a complex linear mixed model. In the GWAS analysis here reported, we analy**z**ed 6,722 high quality SNPs obtained from genotyping a panel of 510 P*. pinaster* plants subjected to an artificial PWN inoculation assay. These SNPs were then used to explore associations with phenotypic variability in PWN response through mixed model theory, aiming to identify useful markers to assist in the selection of PWD-resistant plants.

## 2 Material and methods

### 2.1 Plant material and PWN inoculum

Seeds were collected from five open pollinated *P. pinaster* trees from the reference population for PWD resistance or tolerance (Ribeiro et al., 2012), located at “Herdade da Comporta” (38° 21′ 28.52′′ N, 8° 45′ 49.89′′ W) in Southern Portugal. Three-year-old plants from the five half-sib families (F17, F77, F152, F440, and F465) were used. These families were previously characterized after PWN inoculation of two-year-old plants concerning the estimated survival mean rate, evaluated among 96 families at 157 days post inoculation (dpi), obtained from the fitting of a generalized linear mixed model, as described in (Carrasquinho et al., 2018), resulting in a predicted mean survival in a range of 12.9–25.2% (**Supplementary Table 1**). The necessary permissions were obtained for the collection and use of the seeds, and relevant institutional, national, and international guidelines for plant material collection and experimental work were followed.

The *Bursaphelenchus xylophilus* isolate Bx013.003 used for the inoculation assay and previous assays (Carrasquinho et al., 2018; Modesto et al., 2021, 2022b, 2022a; Rodrigues et al., 2021) is part of the INIAV’s Nematology Laboratory (Oeiras, Portugal) collection and was obtained from an infested tree with wilting symptoms in central Portugal (39°43′33.8′′N, 9°01′55.7′′W). Bx013.003 ITS region sequence is available at GenBank (NCBI) under the accession number MF611984.1. The nematodes were maintained on a non-sporulating *Botrytis cinerea* strain grown on autoclaved barley grains (pure culture) at 25 ± 1°C. Before inoculation, nematodes were allowed to grow on sterilized wood to ensure their virulence. The nematodes were separated from the culture media using the “tray” method (Whitehead and Hemming, 1965) and suspended in water at a concentration of 1,000 nematodes per milliliter.

### 2.2 PWN inoculation assay and symptoms assessment

The artificial PWN inoculation assay was performed in September 2019 on 577 plants maintained in 2,5 L pots in a greenhouse. The plants were arranged according to a completely randomized block design with nine blocks. Within each block, each half-sib family was represented by approximately twelve randomly distributed individuals. The blocks were established to control specific sources of variation, particularly plant vigor (as differences in height and stem diameter had been observed in the nursery) and position inside the greenhouse by grouping plants of similar initial height and diameter within the same block. The height and diameter at the base of the plants were recorded at the beginning of the experiment.

A total of 541 three-year-old plants were inoculated with the PWN following the method of Futai (1979) at mixed developmental stages, while 36 plants were inoculated with water, as a control for symptoms development. Briefly, a suspension with 1000 nematodes was pipetted into a small longitudinal wound made with a sterile scalpel below the apical shoot region in the main stem (simulating the vector feeding). The inoculated wounds were covered with parafilm to prevent the inoculum from drying.

Symptoms were assessed weekly from the 14^th^ dpi until the 447^th^ dpi and were classified according to a visual scale: level 1 – 0% of brown/wilting needles; level 2 – 1-25% of brown/wilting needles; level 3 – 26-50% of brown/wilting needles; level 4 – 51-75% of brown/wilting needles; and level 5 – 76-100% of brown/wilting needles.

To combine all symptom scores into a single value for each plant, the area under disease progress curve (AUDPC) was calculated with the following formula:


$$AUDPC=\;\sum_{i=1}^{N_{i}-1}\frac{\left(y_{i}+\;y_{i+1}\right)}{2}(t_{i+1}-\;t_{i})$$

where $y_{i}$ is the symptom score at time ί, $y_{i+1}\;$is the symptom score at time i+1 and $t_{i+1}-\;t_{i}$ is the number of days between scoring times ί and i+1. Since AUDPC combines disease incidence and time into a unified measure, it can be used to quantitatively summarize the pine wilt disease progression over time and encompass a greater range of phenotypic variance compared to relying only on a single time-point measurement. In simple terms, the lower the AUDPC value, the less susceptible the plant is or *vice versa*. The phenotypic data was deposited in DMPortal from BioData.pt (https://doi.org/10.34636/DMPortal/14N7WM).

As an indicator of plant stress after PWN inoculation, we measured the photosynthetic index of both photosystems I and II (PI_abs_) and maximal efficiency of Photosystem II (Fv/Fm; the ratio of variable to maximum fluorescence after dark adaptation, representing maximum quantum yield of Photosystem II) in a sample of 215 plants before and after inoculation (at 21, 28, 35, 42, 49, 56, 63, 70, 77, 91, 112, and 140 dpi). Spearman’s correlations between the PI_abs_, Fv/Fm values and AUDPC values at the same time-points were calculated using ‘corr’ function in R environment. We also recorded the day/night temperature in the greenhouse during the assay.

### 2.3 DNA extraction and SNP genotyping

Genomic DNA was extracted from needles collected before the inoculation assay using the CTAB method (Doyle and Doyle, 1991) with minor modifications: extraction buffer contained 1% PVP-40, no ammonium acetate was added to the washing buffer, and 0.1 vol. 3M sodium acetate was added to in the final DNA precipitation step. DNA with high quality and integrity, as evaluated by fluorimetry on Qubit and gel electrophoresis, was used for SNP genotyping using a customized SNP array (4TREE Axiom array, B4EST EU-funded H2020 project; Guilbaud et al., 2020, commercially available on demand from ThermoFisher Scientific, USA). This array includes around 50,000 validated SNPs from two *Pinus* (*P. pinea*, P*. pinaster*), two *Populus* and several *Fraxinus* species. The *P. pinaster* SNPs include 12,146 SNPs firstly discovered from an exome capture experiment on a diverse panel of 163 individuals (GenTree project, unpublished) and 3,684 SNPs from a previous Illumina Infinium 9k array (Plomion et al., 2016), that includes fifty-three transcripts displaying differential expression in the presence/absence of PWN infection (Santos et al., 2012). The SNPs are considered highly confident and are located at least 100 bp apart from each other (Guilbaud et al., 2020).

Samples with a dish quality control value ≥ 0.82 (where 1 means no overlap between two homozygous peaks and 0 complete overlap), and call rate ≥ 97% were kept for further analyses. The AXIOM Analysis Suite v.5.1 (Thermo Fisher Scientific Inc., Waltham, MA, USA) was used to obtain genotyping statistics for all samples and SNPs. After eliminating samples with high similarity (> 99.5%) and monomorphic SNPs, a total of 10,823 SNPs (80.73%) and 510 samples (94.3%) were kept for subsequent analyses. Missing genotype data (0.36%) was imputed with the most frequent genotypic class. The preparation and quality auditing of genomic data were conducted using the ASRgwas package in the R environment (Gezan et al., 2022).

### 2.4 Evaluation of plant family susceptibility to PWN inoculation

To evaluate differences among the five families included in the study, data on plant height, basal diameter, and AUDPC at 154, 273, and 447 dpi were analyzed. Analyses were conducted within the framework of linear mixed models (McCulloch et al., 2008). Each trait was analyzed separately.

For height and diameter, the model treated family as a fixed effect factor, block as a random effects factor, and the effects of family×block interaction as random effects. Block effects and family×block interaction effects were assumed to be independent and identically distributed (i.i.d.) random variables with normal distributions, zero mean, and their respective variances. Random errors were also assumed to be i.i.d. normal variables with zero mean and their respective variance. Blocks, family×block interaction effects, and random errors were assumed to be mutually independent.

For AUDPC data at 154, 273, and 447 dpi, separate models were fitted for each time point. These models included family as a fixed effect factor and incorporated height and diameter as covariates. Block was treated as a random effects factor, and the effects of family×block interaction were also modelled as random. Block effects and family×block interaction effects were assumed i.i.d. normal variables with zero mean and their respective variances. For the random errors, a heterogeneous variance structure by block was assumed. Specifically, random errors were considered normal variables with zero mean and block-specific variances, resulting in nine distinct error variance components (block diagonal covariance matrix). Blocks, family×block interaction effects, and random errors were assumed mutually independent.

Variance components were estimated using the residual maximum likelihood (REML) method. Variance components were tested using residual likelihood ratio tests (LRT), applying the conservative approach of assuming that asymptotic distribution of the LRT statistic as chi-squared with one degree of freedom. Fixed effects were tested using a generalized F-test, and multiple comparisons of means were conducted using the Tukey-Kramer method.

All analyses were performed in SAS version 9.4 (SAS Institute Inc, 2016), using the MIXED procedure.

### 2.5 Genome-wide association analysis

To potentially identify SNPs explaining phenotypic variation in the *P. pinaster* response to PWN inoculation (measured by the AUDPC), we applied a linear mixed model using the ‘*ASRgwas*’ package (Gezan et al., 2022), which is used in tandem with *ASReml-R* (Butler et al., 2017). Quality control was performed prior to GWAS removing SNP markers with minor allele frequencies ≤ 0.05, heterozygosity > 0.8, and inbreeding coefficient > 0.98.

GWAS was performed accounting for the phenotypic covariance due to genetic relatedness/kinship and population structure found in the 510-plant panel. To avoid false-positive associations due to correlations rather than true associations caused by relatedness of individuals and/or population structure (Hoffman, 2013; Sul et al., 2018), we introduced both kinship and population structure into the model. The genomic relationship matrix/kinship (G) (additive relationships) was obtained with VanRaden’s method and the population structure matrix (Q) was generated from the G (Patterson et al., 2006). The Q matrix was used to describe the population structure, based on the percentage of explained variance (four dimensions were used, **Supplementary Figure 1**).

GWAS analysis was performed using a linear mixed model to account for all sources of variation affecting AUDPC values, thereby improving the accuracy of the results. To prevent spurious associations, population genetic structure and kinship were included, as described above, along with all other family-related effects (to control for shared genetic markers due to relatedness rather than susceptibility to the pinewood nematode) and relevant experimental design effects. The following linear mixed model was fitted:


$$y=\mu 1+\alpha_{i}s_{i}+X\beta +Q\delta +Zu+e$$

where,

y is a vector of the response variable AUDPC for the n individuals analysed;

μ is the overall mean and 1 a vector of n ones;

$\alpha_{i}$ is the slope associated with the ith marker and $s_{i}$ is the ith column of the marker matrix M;

β is the vector of fixed effects associated with the experimental design (including the effects of the families, blocks, family×block interactions and the diameter as a covariate) and X is the design matrix of the fixed effects;

δ is the vector of fixed population effects and Q is a matrix with vectors describing the population structure;

u is the vector of random additive effects with multinormal distribution with zero mean vector and covariance matrix G (the genomic relationship matrix derived from the markers);

e is the vector of random errors which is assumed to follow a multinormal distribution with zero mean vector and block diagonal covariance matrix (resulting in nine distinct error variance components, one per block of the experimental design).

Significant marker-trait associations were initially identified using a *p*-value threshold of 3.0 x 10–^3^ and Quantile-Quantile (QQ) plot were inspected to assess the distribution of *p*-values. These markers were designated as candidates for a second selection step. After removing collinear markers, a backward selection procedure was applied using a *p*-value threshold of 0.01. At the final step, a Bonferroni correction was applied to the selected markers, using a corrected significance level of α∗=0.05/nselected.

For GWAS analysis, the model with the lowest false discovery rate was chosen. This model considered all the experimental design factors to be fixed, as defined by vector β, and included two random components: one for the genomic effect and the other for the random error.

### 2.6 Estimation of allele effects and inheritance mode

For the SNPs identified as cofactors, we estimated the allele effects in terms of mode of action. Dominance relations among alleles were estimated by studying the phenotypic averages of genotypic classes (homozygous for the reference allele; heterozygous, and homozygous for the alternative allele). Significant differences in AUDPC means among these three genotypic classes were tested by ANOVA and Tukey-Kramer’s Multiple Comparison Tests. The dominance index (h) was then calculated as the ratio between the deviation of the heterozygote mean from the lower homozygote mean and the difference between the two homozygote means:


$$h=\frac{\left(Mean_{AB}-Mean_{AA}\right)}{\left(Mean_{BB}-\;Mean_{AA}\right)}$$

where,

AA represent the homozygous genotypes for the reference allele;

BB represent the homozygous genotypes for the alternative allele;

AB represents the heterozygous genotype.

Linkage disequilibrium (LD) among significant SNPs was estimated using genotype-based *r²* calculated from additive genotype scores (0, 1, and 2) in the R environment.

To assess epistatic effects among the six selected SNPs, pairwise interactions were tested using separate mixed linear models in *ASReml* (Butler et al., 2017), with the corresponding SNPs and their interaction as fixed effects. The significance of each interaction was evaluated using Wald tests, and p-values were adjusted for multiple testing using the Bonferroni correction (α = 0.05/15 pairwise comparisons).

### 2.7 SNP mapping into candidate genes

Since no reference genome is available for *P. pinaster*, the location of the identified SNPs was investigated by a similarity search of the SNP flanking regions against the annotated *P. pinaster* transcriptome (Modesto et al., 2021). BLASTn (BLAST+ v2.2.31) (Camacho et al., 2009) alignments were performed with a minimum *E*-value of 1e-3, and the best hit (lower *E*-value) was retained for each SNP. The differential expression of the identified transcripts was investigated in the available transcriptional data (Modesto et al., 2021). Although the population in the referred paper differed from the GWAS one, the analysed plants belonged to a family represented in the GWAS, enabling biologically meaningful comparison. Functional annotation of the SNPs was performed using SnpEff v4.3t (Cingolani et al., 2012).

## 3 Results

**Figure 1:**
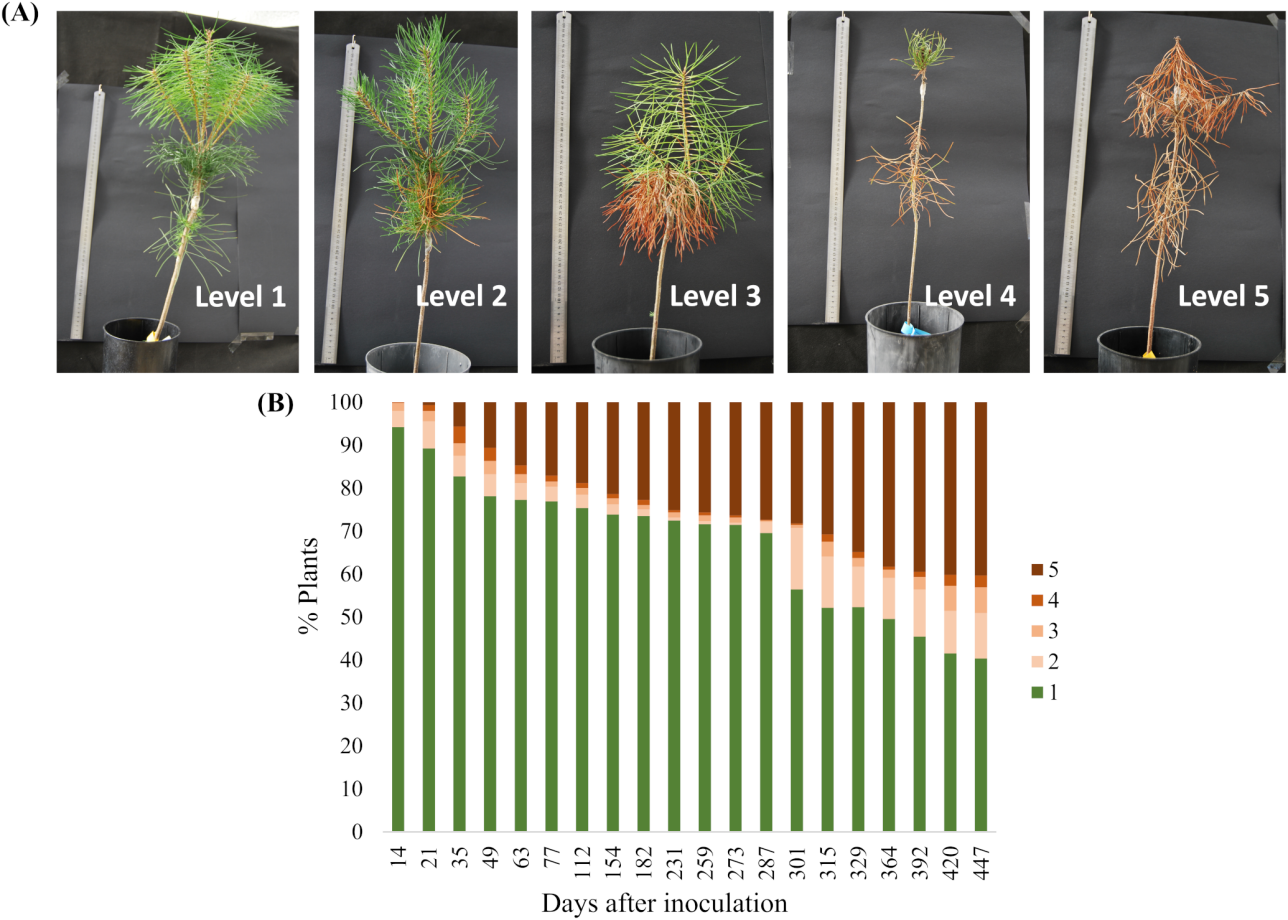
Progression of pine wilt disease in the inoculation assay. **(A)** Disease severity visual scale: 1 – 0% of brown/wilting needles; 2 – 1-25% of brown/wilting needles; 3 – 26-50% of brown/wilting needles; 4 – 51-75% of brown/wilting needles; and 5 – 76-100% of brown/wilting needles **(B)** Symptoms progression after PWN inoculation based on the disease severity visual scale.

The first symptoms of progressive wilting appeared 14 dpi with PWN (levels 2-5, **Figure 1**). By the end of the assay, 59.7% of the plants presented symptoms (levels 2-5, susceptible plants, **Figure 1**), while 40.3% showed no symptoms (level 1, resistant plants, **Figure 1**). The plant survival at the end of the trial was 50.9%. Plants considered resistant may in fact be tolerant, maintaining a healthy phenotype despite PWN multiplication (Trudgill, 1991; Woodcock et al., 2018), since disease classification relied exclusively on a visual external symptoms rather than nematode quantification, which was not assessed in this study. Nevertheless, true resistance, defined as the ability to inhibit PWN multiplication, has been reported in other *P. pinaster* families (Menéndez-Gutiérrez et al., 2018). During the assay, the photosynthetic index of both photosystems I and II (PI_abs_) and maximal efficiency of Photosystem II (Fv/Fm) were negatively correlated with AUDPC after inoculation, particularly from 49 dpi onwards (Spearman’s correlation coefficients > 0.5, **Supplementary Figure 2**), indicating reduced photosynthetic performance and increased plant stress.

### 3.1 Evaluation of plant family susceptibility to PWN inoculation

Significant differences among the five families were detected in basal diameter, whereas no evidence was found to reject the null hypothesis for total height (**Supplementary Table 2**). Family F17 exhibited the largest mean basal diameter, which was significantly higher than that of families F440 and F152 (**Supplementary Figure 3**). No significant differences were observed among the mean basal diameters of families F77, F152, F440, and F465 (**Supplementary Figure 3**).

Considering the experimental design, the block variability was significant (p < 0.01), indicating heterogeneous environmental conditions within the greenhouse. In fact, the empirical best linear unbiased predictors (EBLUPs) of the block effect for plant diameter at the base (EBLUP_d) and total height (EBLUP_h) showed that the predicted effects of the block ranged from -0.4733 to +0.8708 mm for diameter and from -6.5284 to +9.1939 cm for height, relative to the trial mean (**Supplementary Table 3**). Significant variability for family×block interaction was also detected (p < 0.01) (**Supplementary Table 2**).

The AUDPC values measured at 154, 273, and 447-days post-inoculation (dpi) ranged from 238.48 to 939.64 (**Table 1**). The effects of basal diameter and total height on AUDPC varied depending on dpi. However, at a significance level of 0.01, the effect of both covariates was not significant (**Table 1**). Regarding the experimental design effects on AUDPC, block variability was not significant (*p* > 0.01), as well as for family×block interaction variability (*p* > 0.01), indicating that families behaved consistently across blocks (**Table 1**).

**Table 1:** Results from fitting the linear mixed model for the area under the disease progress curve (AUDPC) calculated at 154, 273, and 447 days post inoculation (dpi): overall phenotypic mean; F-test for the fixed effects of the family factor and the corresponding p-values; estimated coefficients for the covariates diameter at the base ( ${\hat{\beta }}_{1}$) and height ( ${\hat{\beta }}_{2}$), along with the p-values from the T-tests for the corresponding parameters; block variance estimate $({\hat{\sigma }}_{Block}^{2}$) and family×block interaction variance estimate ( ${\hat{\sigma }}_{Fam\times Block}^{2}$) and their respective p-values for testing the corresponding variance components.

dpi	AUDPC overall mean	F value Family effects (p-value)	${\hat{\beta }}_{1}$ (p-value)	${\hat{\beta }}_{2}\;$(p-value)	${\hat{\sigma }}_{Block}^{2}$ (p-value)	${\hat{\sigma }}_{Fam\times Block}^{2}$(p-value)
154	238.48	4.60 (0.0050)	-11.17 (0.0797)	-1.65 (0.1188)	5573.38 (0.0367)	712.01 (0.1412)
273	478.96	5.55 (0.0017)	-29.23 (0.0269)	-3.65(0.0992)	23051 (0.0372)	3177.71 (0.1386)
447	939.64	8.07 (0.0001)	-35.75 (0.1337)	-9.67 (0.0120)	55886 (0.0401)	7781.89 (0.1847)

Significant family effects on susceptibility to pine wilt disease (PWD) were observed at all evaluation dates post-inoculation (*p* < 0.01), with the magnitude of these effects increasing over time. This effect was largely attributable to family F440. Multiple pairwise comparisons showed that the mean AUDPC at 154 and 273 dpi differed significantly between F440 and families F465 and F77 (**Supplementary Figures 4**, **5**). At 447 dpi, the mean AUDPC of family F440 differed significantly from all the others under study (**Supplementary Figure 6**). No significant differences were observed among the families F17, F77, F152, and F465 at any of the three evaluation dates.

For GWAS analysis, AUDPC data at 447 dpi was used, as these measurements exhibited the most pronounced differences in the susceptibility among families.

### 3.2 Identification of candidate *loci* associated with PWN response

The genotyping of the 510-plant panel yielded 10,823 high-confidence SNPs, including 8,709 polymorphic and 2,114 monomorphic markers. Stringent filtering removed 1,987 additional markers: 1,893 SNPs with MAF <5%; 91 with heterozygosity > 80%; and three with inbreeding coefficient >98% (**Supplementary Figure 7**). This process resulted in a curated set of 6,722 high-confidence SNPs to be used for the GWAS analysis. Detailed information, including flanking sequences, as well as reference and alternative alleles, is provided in **Supplementary Table 4**.

With the fitting of the linear mixed model, a total of 26 markers were identified using a p-value threshold of 0.003. The observed false discovery rate (FDR) for this set was 0.7756, indicating that only about 6–7 of these markers are likely to represent true associations. After applying the backward marker selection procedure, the set of candidate SNPs was reduced to seven, of which six remained significant after Bonferroni correction (**Table 2**).

**Table 2:** Significant SNP markers with respective minor allele frequency (MAF), reference and alternative allele, SNP effect on the area under the disease progress curve (AUDPC), and percentage of explained genetic variance.

SNP marker	MAF	Ref allele	Alt allele	SNP effect	Std. error	Z.ratio	P-value	Expl. var
AX-366099317	0.33137255	C	A	213.1577	73.26444	2.909429	1.12E-03	4.833693
AX-366108903	0.36470588	A	G	278.9363	84.53337	3.299718	4.46E-04	8.655733
AX-366109081	0.06764706	T	C	560.0849	134.056	4.177993	6.56E-06	9.499773
AX-365965826	0.47156863	C	G	-229.364	78.91338	-2.90653	1.29E-03	6.294474
AX-366030902	0.45490196	G	A	-225.176	59.18963	-3.80431	4.22E-05	6.036873
AX-365972576	0.1372549	A	C	331.1084	88.79391	3.728954	1.10E-04	6.233430

Significant SNP markers detected in the final analysis using the Bonferroni-corrected significance level (α* = 0.007142857).

The six SNPs account individually for about 4.8 – 9.50% of the additive genetic variance on AUDPC, and collectively explained up to 41.6% of the total phenotypic variance (**Table 2**).

**Figure 2:**
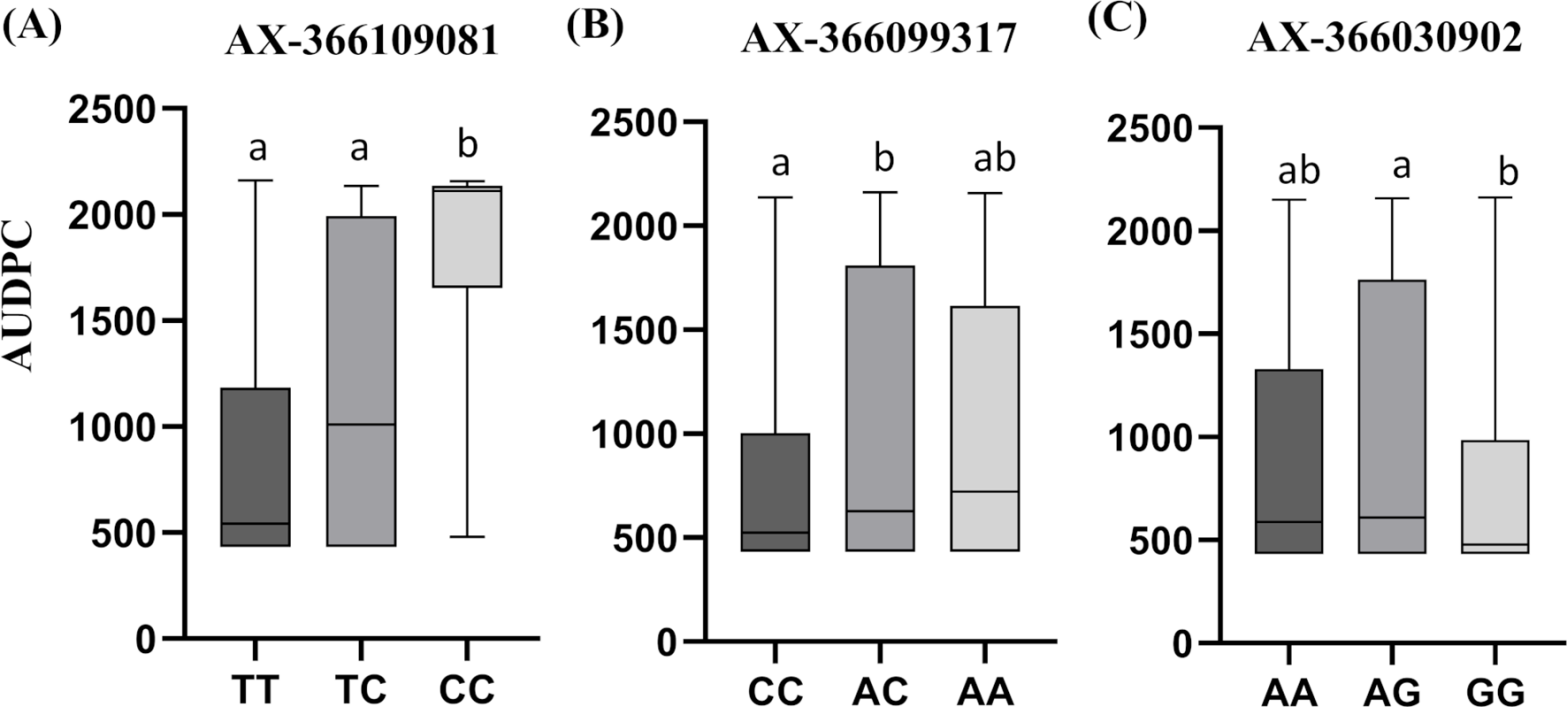
Boxplots of three significant SNPs where the effect of alleles on AUDPC can be observed. **(A)** The C allele on AX-366109081 *locus* is partially dominant (h = 0.34) and associated with higher AUDPC values (increased susceptibility to PWN), and the T allele with lower AUDPC (potential resistance). **(B)** The heterozygous genotype (AC) on AX-366099317 SNP marker shows the highest AUDPC and is significantly more susceptible than the homozygous CC genotype, indicating underdominance (h = 1.37) **(C)**. The GG homozygous genotype on AX-366030902 *locus* shows the lowest AUDPC and is significantly different from the heterozygous AG genotype, indicating underdominance (h = 1.74). h = dominance index estimation.

Three of the detected SNPs also showed significant effects in the ANOVA single marker analysis, supporting their role in the mechanisms underlying the response to PWN. Marker AX-366109081 showed the largest effect, accounting for 9.5% of the additive genetic variance. However, similarity searches against the *P. pinaster* transcriptome failed to retrieve any significant hit; thus, no functional annotation or candidate gene could be assigned to this locus. For AX-366109081, the C allele was associated with higher AUDPC values (increased susceptibility to PWN), and the T allele with lower AUDPC values (potential resistance). Genotype means followed the pattern CC > TC > TT (**Figure 2A**). The dominance index estimation (h = 0.34) based on genotype means (CC = 1839, TC = 1214, TT = 894) suggests a partially dominant effect of the C allele. This is supported by the Tukey-Kramer’s test, which showed that the susceptible homozygote (CC) and the heterozygote (TC) were not significantly different (*p* = 0.0559), while both were significantly higher than the resistant homozygote (TT; *p* ≤ 0.001).

The second SNP showing significant allelic effects was AX-366099317 (*p* = 0.0085; ANOVA), located in *carboxyl-terminal-processing peptidase 1* (*CTPA1*), a gene involved in chloroplast function and photosynthesis (Oelmüller et al., 1996). The heterozygous genotype (AC) showed the highest disease progression (mean AUDPC = 1032), significantly higher than the CC homozygote (mean = 846, *p* = 0.0067, **Figure 2B**). The AA genotype also showed elevated AUDPC values (mean = 981), but differences were not statistically significant compared to other classes, likely due to the reduced sample size of this group [n=62 versus AC (n = 214) and CC (n = 234)]. This SNP showed a dominance index of 1.37 when considering C as the reference allele, indicating overdominance. This pattern suggests heterozygote disadvantage, where heterozygous individuals exhibit greater disease progression than either homozygote.

The third SNP, AX-366030902 (*p* = 0.0362, ANOVA) is located in *phosphatidylinositol 4-phosphate 5-kinase 9* (*PIP5K9*), encoding a key enzyme in the phosphoinositide signalling pathway (Zarreen et al., 2023). The GG homozygous genotype was associated with the lowest AUDPC, differing significantly from the heterozygous AG genotype (*p* = 0.0284, **Figure 2C**). This SNP also showed a overdominance effect (h = 1.74) when considering G as the reference allele. The remaining three identified SNPs (AX-366108903, mapped on *40S ribosomal protein S26-1, RPS26*; AX-365965826 (mapped on *oxygen-evolving enhancer protein 2, OEE2*, and AX-365972576, located on *enhanced disease susceptibility 1-like protein, EDS1L*; **Table 3**), were significant in the GWAS, but not in the single-marker ANOVA (p>0.05; **Supplementary Figure 8**). The discrepancies between the GWAS and the ANOVA analysis suggest the effects of these *loci* are likely small, dependent on the genetic background or masked by population structure (or other design effects) in simpler statistical models such as one-way fixed effects ANOVA.

**Table 3:** SNP variant type, predicted gene annotation and protein domains.

SNP marker	Variant type	Annotation	Interpro domain description
AX-366099317	5’ UTR	Carboxyl-terminal-processing peptidase 1, chloroplastic	PDZ domain|C-terminal-processing peptidase S41A|Tail specific protease|ClpP/crotonase-like domain superfamily|PDZ superfamily|PDZ domain 6
AX-366108903	Synonymous	40S ribosomal protein S26-1	Ribosomal protein S26e superfamily
AX-366109081	NA	NA	
AX-365965826	5’ UTR	Oxygen-evolving enhancer protein 2, chloroplastic-like	Nonaspanin (TM9SF)
AX-366030902	Synonymous	Phosphatidylinositol 4-phosphate 5-kinase 9	Phosphatidylinositol-4-phosphate 5-kinase, core|MORN motif|
AX-365972576	Missense	Protein EDS1L-like	Fungal lipase-like domain|Alpha/Beta hydrolase fold|EDS1, EP domain

Variant prediction identified one missense, two synonymous, and two 5’-UTR variants (**Table 3**). Most markers (83.3%) were detected in transcripts with predicted protein annotations, including families associated with biotic stress and signalling pathways: EDS1L, RPS26, PIP5K9, DHQS, CTPA1, and OEE2 (**Table 3**).

Analyzing the expression results presented in Modesto et al. (2021), the transcripts *DHQS* and *OEE2* were slightly upregulated after inoculation, both in susceptible and resistant plants, while *EDS1L* was slightly upregulated only in susceptible *P. pinaster* [Log2(fold change) = 0.21, **Supplementary Table 5**, Modesto et al., 2021]. On the other hand, *CTPA1* was downregulated, while *RPS26* and *PIP5K9* were not differentially expressed after inoculation (**Supplementary Table 5**).

### 3.3 Two SNPs show significant epistatic interaction

**Figure 3:**
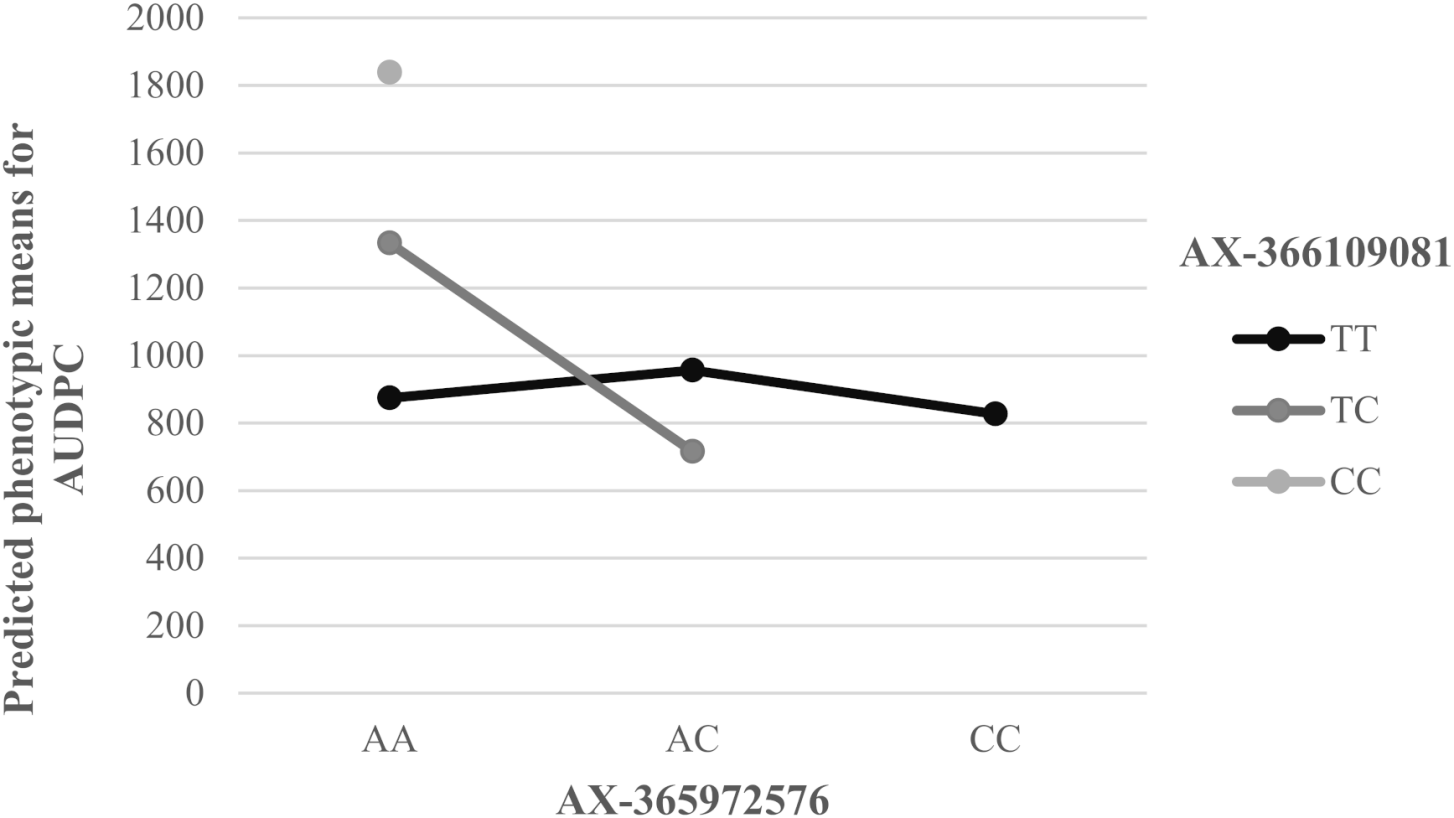
Interaction plot of model-predicted phenotypic means for the area under the disease progress curve (AUDPC). The predicted mean values were obtained by fitting a linear mixed model including the fixed effects of the two SNPs (AX-366109081 and AX-365972576) and their interaction.

To further explore the genetic architecture of PWD resistance, we tested for pairwise epistatic interactions among the six most significant SNPs detected. Interestingly, a single significant interaction was detected between the unannotated locus AX-366109081 and the *EDS1L-like* missense variant AX-365972576 (p-value = 0.0016, **Supplementary Table 6**). This interaction indicates a non-additive effect on AUDPC, where the phenotypic impact of one locus is modulated by the genetic background of the other. As evidenced in the interaction plot of model-predicted phenotypic means (**Figure 3**), a strong synergistic effect was observed: the double heterozygous genotype (TC/AC) resulted in a greater-than-additive reduction in AUDPC (interaction effect of -699.32, **Supplementary Table 7**), significantly increasing resistance (**Supplementary Table 8**). This synergistic response is particularly evident when comparing the TC/AC genotype with other combinations; for instance, the presence of the C allele in AX-366109081 leads to a sharp increase in susceptibility unless it is compensated by the AC configuration at the interacting locus. These two SNPs showed very low genotype-based LD among them (*r²* = 0.007) as observed for the remaining SNPs, which was uniformly low (*r²* < 0.04) (**Supplementary Table 9**), confirming that the interaction reflects biological epistasis rather than physical linkage. No other pairwise combinations showed significant epistasis, indicating that while the architecture is predominantly additive, specific non-linear interactions also contribute to the trait’s variation. The absence of certain allelic combinations in our analysis (indicated in **Supplementary Table 8**) was expected, as the available genotypic space was limited by the pedigree structure of our half-sib families, a common feature in forest genetic studies.

## 4 Discussion

Pine wilt disease remains a persistent and serious threat to *P. pinaster* forests, and no effective breeding program has been developed so far. Previous studies have demonstrated the genetic variability of *P. pinaster* in the response to PWN infection (Carrasquinho et al., 2018; Menéndez-Gutiérrez et al., 2018), uncovering the potential for an effective breeding program. In this study, we performed a PWN inoculation assay to identify genomic variants associated with the phenotypic response to PWN, that could be potentially used for marker-assisted selection.

The survival rate of the inoculated plants at the end of the assay (around 51%) is consistent with the results from other artificial inoculation assays carried out in *P. pinaster* under comparable plant developmental stage, day/night temperatures and growth conditions (63-71%) (Menéndez-Gutiérrez et al., 2018), albeit it was reached over a longer period in our study. Although the inoculum size was doubled in the present work compared with the above referred assay, the PWN inoculation in mid-September and lower nocturnal temperatures might have affected the speed of PWN progression. Maximum mortality was reached when mean temperature was over 20 °C, with night temperature showing greater influence on mortality (Menéndez-Gutiérrez et al., 2017). Moreover, a seasonal pattern in plant mortality has been observed with higher seedling mortality rates found for March inoculations when compared with other months under the same climatic conditions in *Pinus sylvestris* and *Pinus contorta* (Panesar and Sutherland, 1989).

Higher stem diameter and more branches were previously correlated with increasing survival after PWN inoculation (Yamanobe, 2009; Carrasquinho et al., 2018). In this study, an indirect effect of diameter on AUDPC values and, consequently, on susceptibility to PWN was detected through the family effect. Indeed, family F440 showed a significantly lower mean diameter and higher susceptibility compared with family F17. Interestingly, in the F440 family there are more individuals with the allele associated with susceptibility in all SNP markers identified in this study. In conifers, genetic variation in resistance to other biotic stressors has been documented across different systems and often exhibits significant genetic variation among families or populations (Zas et al., 2005, 2017; Moreira et al., 2013). A recent GWAS in *Picea abies* identified 12 SNPs associated with survival during a severe bark beetle outbreak, demonstrating a heritable component of resistance in natural populations (Korecký et al., 2023). Additionally, association genetics in white spruce linked defence metabolite variation to constitutive insect defence phenotypes (Lamara et al., 2018).

Although the six identified SNPs explained individually moderate additive genetic variance from 4.8 – 9.50%, their cumulative effect (41.6%) suggest a polygenic architecture for the genetic control of PWN plant response, with no large effect alleles segregating in the 510-plant panel analyzed. This complexity contrasts with the major locus (27%) for resistance found in the most PWN resistant variety of *P. thunbergii* (Hirao et al., 2019, 2022) but is consistent with more than one century of evidence demonstrating that many heritable quantitative traits follow a polygenic architecture, being influenced by a large number of *loci* with small effects (Barton et al., 2017; Galton, 1877; Fisher, 1956; Younessi-Hamzekhanlu and Gailing, 2022; Demirjian et al., 2023). The absence of large effect alleles may also be related with the low marker density (6,722 SNPs) relative to the approximately 28 Gb *P. pinaster* genome (Sterck et al., 2022), which likely have prevented a comprehensive representation of the trait variation. This limitation, combined with the rapid LD decay in *P. pinaster* (Plomion et al., 2014), underscores the need for higher-density genotyping or sequencing approaches. A rapid LD decay enables high-resolution mapping but also requires high marker density. Interestingly, we detected a significant epistatic interaction between two statistically independent *loci*, suggesting the presence of non-additive genetic effects instead of a correlation between linked *loci*. However, given the moderate sample size, rapid LD decay and limited marker coverage, further validation will be necessary to confirm the robustness and biological basis of this interaction.

The SNPs identified in this study can also be in LD with the causative variants rather than being causal themselves. In many cases, the true causal variant is an ungenotyped structural variant close to numerous SNPs, which are inherited together and therefore appear statistically associated with the phenotype, due to the formation of LD blocks (Clauw et al., 2025). Long-range LD can also generate significant associations through epistatic interactions between loci that are not physically linked (Liu et al., 2025a). Although LD in *P. pinaster* decays rapidly over short distances (Plomion et al., 2014), it can vary depending on recombination and population history. The functional classes predicted for the identified SNPs (missense, synonymous, and 5’UTR variants) suggest multiple regulatory layers. Missense polymorphisms (single amino-acid substitutions) that have been associated with disease resistance can alter protein function (reviewed in Marchal et al., 2022; Contreras et al., 2023). Synonymous or silent variants can affect post-transcriptional processes like alternative splicing, alternative polyadenylation or alternative initiation with regulatory roles in plant immunity (Thieffry et al., 2022; reviewed in Zhou and Li, 2023; Lin et al., 2024; Alhabsi et al., 2025). Also, 5’UTR variants are a well-established layer of post-transcriptional control of plant immunity by impacting mRNA stability, ORF number or reading frame, RNA secondary structure, transcription start site selection and promoter strength, translation efficiency, among others (reviewed in Xiang and Dong, 2025). Our findings are consistent with the polygenic nature of the disease resistance and emphasize the intricate genetic mechanisms governing response to PWN.

As stated above, *P. pinaster* has a highly heterozygous diploid genome of approximately 28 Gb distributed in 12 chromosomes, with a large percentage of repeated regions (Sterck et al., 2022). Although major efforts have been made so far to make available a high-quality genome of *P. pinaster*, its complex assembly remains a significant obstacle. Identifying genetic variants underlying phenotypic variation through GWAS without an available reference genome is possible but with some limitations (Voichek and Weigel, 2020). Since SNPs are not necessarily the causal variant themselves, the detection of the genomic regions upstream and downstream the significant SNPs is needed to identify candidate genes. The distance between a significant SNP and a causal gene or genomic region can vary greatly, from small (2 kb) to large (up to 500 kb), depending on several factors including genome size and complexity, the rate of LD decay and the availability and density of genetic markers as previously mentioned. In this work, we used the annotated transcriptome of *P. pinaster* to partially overcome the absence of an annotated genome, but bearing in mind that the precise location of the SNP markers could not always be identified due, for example, to low or absent expression of certain genes in the transcriptome used, or because the SNPs were located in non-transcribed regions.

Notably, we identified SNPs predicted to localize in coding regions of genes or gene families previously related to plant biotic stress responses. For instance, one SNP marker is localized in a putative *EDS1* gene, which is a master regulator of salicylic acid (SA)-mediated immunity (Dongus and Parker, 2021). EDS1 forms complexes with SAG101 or PAD4 proteins, activating distinct defence responses. EDS1-SAG101 dimers activate effector-triggered immunity mediated by TNL (interleukin-1 receptor-like (TIR)-NB-LRR) receptors, while EDS1-PAD4 promote basal immune responses that can be initiated by NLRs or cell surface receptor proteins (Dongus and Parker, 2021). Although the transcript in which the SNP was found was only marginally upregulated in susceptible plants [Log2(fold change) = 0.21] (Modesto et al., 2021), other transcripts with the annotation of *EDS1* were more upregulated, especially in susceptible plants [Log2(fold change) susceptible plants = 2.8 ± 0.12; Log2(fold change) resistant plants = 1.78 ± 0.07] (Modesto et al., 2021). Interestingly, the expression of genes that encode for proteins which physically interact with EDS1 were also differentially expressed, including *SAG101* (upregulated; susceptible > resistant) and *PAD4* (upregulated; susceptible > resistant), as well as the NLR receptors *ADR1* (upregulated) and *SNC1* (downregulated in susceptible), which interact with the EDS1-PAD4 dimer (Dongus and Parker, 2021). These differences in gene expression 72h after inoculation indicate a significant role of EDS1 mediated defence responses in PWN-*P. pinaster* interaction. In fact, the same work shows an accumulation of SA only in susceptible plants, suggesting that the activation of SA-mediated immunity may be relevant for susceptibility. As the SNP detected in the current work is a missense variation, leading to an alteration in the protein sequence, it may impact the interaction with PAD4 and SAG101 proteins, or the activation of these dimers by receptors, such as ADR1 and SNC1, affecting the downstream activation of defence response genes. Moreover, a *EDS1-like* transcript was predicted as target for two different miRNAs expressed in PWN inoculated plants (Modesto et al., 2022b). These results suggest that the *EDS1-like* variant here described might have a functional role in susceptibility to PWD and is an interesting candidate gene for further analysis. Moreover, the detection of a robust epistatic interaction between this variant and the unannotated AX-366109081 is particularly relevant because it suggests that the ‘resistant’ allele at the *EDS1L locus* may require a specific genetic background at AX-366109081 *locus* to fully manifest its protective effect against the nematode. Although the mapping of this *locus* remains uncertain, its location within a non-coding regulatory region involved in the PWD response is plausible.

A synonymous SNP marker was predicted to be localized in *RPS26*, encoding a 40S ribosomal protein S26-1. Ribosomal proteins have been proposed as targets of pathogen effectors in different plant-pathogen interactions. For instance, the obligate biotroph *Blumeria graminis* (causing powdery mildew) effector protein CSEP0064/BEC1054 binds to the host ribosomes inhibiting ribosome-inactivating proteins (RIPs) to likely overcome the host cell death repressing plant immunity (Pennington et al., 2019). Also, the near-obligate pathogen *Phytophthora infestans* effector Pi23226 was shown to interact with ribosomes inducing ribosome malfunction in host cells to induce necrotrophic cell death and enhancing *P. infestans* pathogenicity likely through hijacking essential host cell components from the dying tissue (Lee et al., 2023). Recently, Liu et al. (2025b) reported cellular co-localization of the potato 40S ribosomal protein StRPS5 and *P. infestans* Pi16275 effector. Silencing of *StRPS5* leads to increased susceptibility and decreased levels of stress signalling reactive oxygen species (ROS), whereas its overexpression enhanced disease resistance and ROS accumulation, suggesting that StRPS5 is an Pi16275 effector target, positively regulating resistance by increasing the accumulation of ROS. The transient expression of *Citrus* RPSA-2 in *Nicotiana benthamiana* also triggered signalling pathways such as the JA, and resistance-related genes, with ClRPSA-2 negatively regulating citrus yellow vein clearing virus resistance (Liao et al., 2025). Since a transcript annotated as 40S ribosomal protein S16-A was upregulated in both *P. pinaster* susceptible and resistant plants when compared to controls (Modesto et al., 2021), it is reasonable to hypothesize that 40S ribosomal genes are important in *P. pinaster* response to PWN. Furthermore, transcripts annotated as 40S ribosomal proteins, including a *RPS26* transcript, were predicted as targets of miRNAs expressed in PWN inoculated plants and PWN miRNAs (Modesto et al., 2022b). Therefore, it is possible that the SNP here detected in *RPS26* may influence the post-transcriptional regulation of this transcript or even its targeting by PWN miRNAs, affecting protein translation. This variant could therefore be of functional relevance for resistance to PWN, even though it does not change the protein sequence, and should be further investigated.

A synonymous SNP in the coding region of a putative *PIP5K9* is also interesting, as PIP5Ks were shown to be involved in plant-pathogen interactions (Ivanov and Harrison, 2019; Menzel et al., 2019; Shimada et al., 2019; Qin et al., 2020; Khamesa-Israelov et al., 2024). PIP5Ks are primarily localized to the plasma membrane and synthesize the signalling phospholipid phosphatidylinositol (4,5)-bisphosphate (PI(4,5)P2), a phosphoinositide that regulates signal transduction pathways leading to abiotic and biotic stress resistance (Zarreen et al., 2023). It was recently shown that an Arabidopsis line carrying an artificial microRNA targeting PIP5K9 and PIP5K7 is tolerant to *Pseudomonas syringae* but susceptible to *Botrytis cinerea* (Khamesa-Israelov et al., 2024). Also, the lower levels of PI(4,5)P2 in *pip5k1pip5k2* double mutants inhibited the development of powdery mildew fungus *Erysiphe cichoracearum* and caused disease resistance (Qin et al., 2020) while *pip5k4* and *pip5k6 Lotus japonicus* mutants showed increased rhizobial infection (Akamatsu et al., 2025). In Arabidopsis, the enzymatic activity of PIP5K6 was shown to be suppressed by the signalling of pathogen-associated molecular patterns (PAMPs) through a mitogen-activated protein kinase (MAPK) cascade upon flg22 treatment (Menzel et al., 2019). In *P. pinaster*, genetic variation in *PIP5K* could lead to differential PI(4,5)P2 accumulation resulting in differential stress signalling upon PWN infection.

Two other SNPs mapped to chloroplast-related genes (*OEE2*, and *CTPA1*). The OEE2 protein is a core component of the photosystem II (PSII) (Yi et al., 2007) and CTPA1 is a serine-type protease essential to produce the reaction center protein of PSII and photosynthesis (Che et al., 2013). The role of chloroplasts is critical for plant immunity, where they act as sensors of environmental changes and key regulators of defence responses to pathogens (Rui et al., 2025). Chloroplasts produce ROS, cytoplasmic Ca^2+^ waves, precursors of hormones such as JA, SA and abscisic acid (ABA), alkaloids, lignin, and phenylpropanoids (Sun et al., 2025), all shown to be activated after PWN inoculation in *P. pinaster* (Gaspar et al., 2017; Rodrigues et al., 2021; Modesto et al., 2022c). Chloroplast processes are also direct or indirect targets of pathogen effectors to suppress immunity (Littlejohn et al., 2021). Indeed, the OEE2 protein (also known as PsbP), was shown to be targeted by a *Plasmopara viticola* effector to inhibit ROS production in grapevine (Liu et al., 2021). In Arabidopsis, OEE2 is phosphorylated by cell-surface receptor kinases WAK1 upon treatment with avirulent *Pseudomonas syringae* (Yang et al., 2003). Both *OEE2* and *CTPA1* were slightly upregulated after inoculation, supporting a possible role in *P. pinaster* defence response to PWN. Genetic variation in *P. pinaster* chloroplast-related genes could lead to differential stress signalling. A notable finding was the detection of overdominance at the AX-366099317 (*CTPA1*) and AX-366030902 (*PIP5K9*) *loci*, where heterozygous individuals exhibited higher disease progression than either homozygote. Given that *B. xylophilus* is an invasive parasite, *P. pinaster* genotypes conferring susceptibility have not been subjected to long-term negative selection, allowing such non-additive effects to persist at high frequencies. While true overdominance is relatively rare, this pattern may have been caused by other selective pressures or be explained by pseudo-overdominance (Birchler et al., 2010). This phenomenon is common in genomic regions with low recombination and can be created by the complementation of deleterious alleles in the heterozygotes or by causal variants in repulsion phase (Salson et al., 2025). It is also possible that these markers are in different linkage phases with the true causal variant across different families, considering that our study is based on five half-sib families. This “flip-flop” effect can artificially create a signal of overdominance when data are pooled, as the same marker allele may be associated with both resistance and susceptibility depending on the genetic background.

While overdominance at *CTPA1* and *PIP5K9 loci* was associated with increased susceptibility, the interaction between EDS1L and AX-366109081 revealed a different pattern, where allelic specific combinations enhance resistance. These results suggest that what might appear as overdominance at a single locus is actually part of a coordinated genetic network, where allelic specific configurations are required to trigger an effective defense response. This underscores that resistance is not merely the sum of independent genetic effects but the result of convergent metabolic and immune signalling.

## 5 Conclusions

This study demonstrates that GWAS is a valuable approach for uncovering genetic factors associated with susceptibility to PWN in *Pinus pinaster*. However, the polygenic nature of this trait, combined with this species huge genome and rapid LD decay, underscores the need for large population sizes and higher marker densities or sequencing approaches to capture a broader range of genetic variation. This should be combined with advanced statistical models to improve the resolution of association signals and facilitate the identification of causal variants. Although precise localization of causal variants remains challenging, this work highlights candidate *loci* linked to chloroplast function and immune signalling pathways, providing targets for future functional validation.

The non-additive genetic effects identified provide a mechanistic explanation for some the ‘missing heritability’ often encountered in forest tree GWAS and underscores the complexity of PWN response and the importance of considering them in future breeding strategies. This has practical implications for Marker-Assisted Selection (MAS) and the development of resistant varieties. In most breeding programs, selection is based on the cumulative effect of individual loci. However, our results suggest that for the *P. pinaster*–PWN pathosystem, a more effective strategy would be to prioritize specific multi-locus combinations rather than individual SNPs, as the protective effect of genes like *EDS1L* may depend on the complementary genetic background at interacting *loci*. Therefore, incorporating these non-linear interactions into genomic prediction models could enhance the precision of early selection, allowing for more reliable identification of elite genotypes with enhanced resilience to pine wilt disease.

After rigorous validation in independent populations and families, assessment of allelic effects across genetic backgrounds, and evaluation of marker stability across environments, the SNP markers identified here hold promise for integration into MAS strategies. This would enable the early identification of resistant genotypes for afforestation in maritime pine breeding programs.

## Data Availability

The datasets presented in this study can be found in online repositories. The names of the repository/repositories and accession number(s) can be found in the article/**Supplementary Material**.
