# Supplementary material for: Genome-wide association study reveals genomic regions associated with Pinus pinaster response to pinewood nematode
Source: Front Plant Sci. 2026 Mar 13;17:1765158. doi: 10.3389/fpls.2026.1765158 (PMC13021666; doi:10.3389/fpls.2026.1765158)
Supplement: Supplementary file 2 [file DataSheet1.pdf]

Supplementary data

Genome-Wide Association Study reveals genomic regions associated with *Pinus pinaster* response to pinewood nematode

Vera Inácio, Inês Modesto, Elsa Gonçalves, Ana Vila Verde, Ana Milhinhos, José Antonio Cabezas, María Teresa Cervera, Isabel Carrasquinho and Célia M Miguel

Supplementary Table 1. Estimated mean survival rate for the five families studied at 157 days post-inoculation, and their respective ranking amongst the 96 families studied in Carrasquinho et al. (2018).

Families	Survival rate	
	%	Ranking
F17	12.9	33
F77	20.7	3
F152	25.2	1
F440	15.0	15
F465	15.2	14

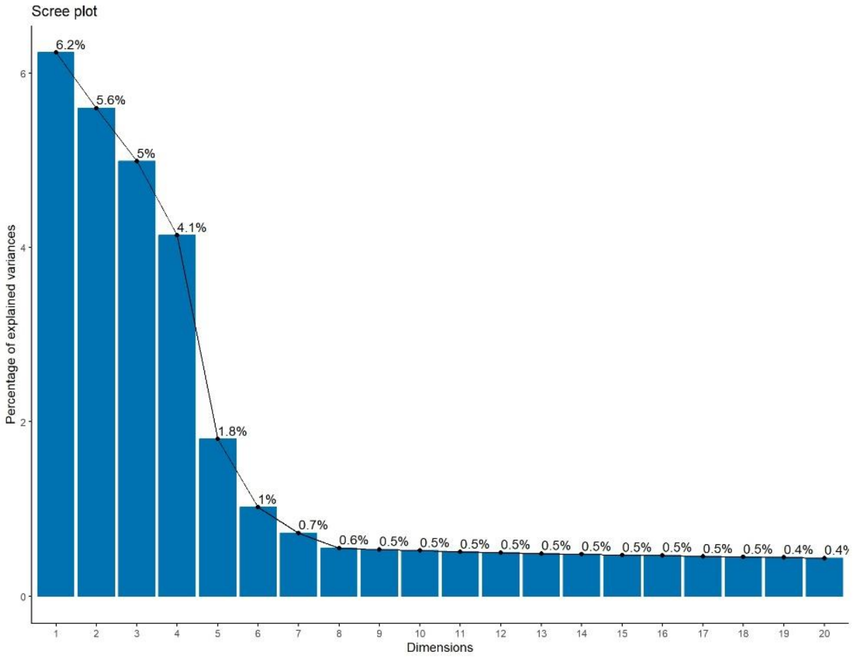

Supplementary Figure 1 - Scree plot of the explained variance in the area under the disease progress curve (AUDPC) of the first 20 dimensions obtained from principal component analysis.

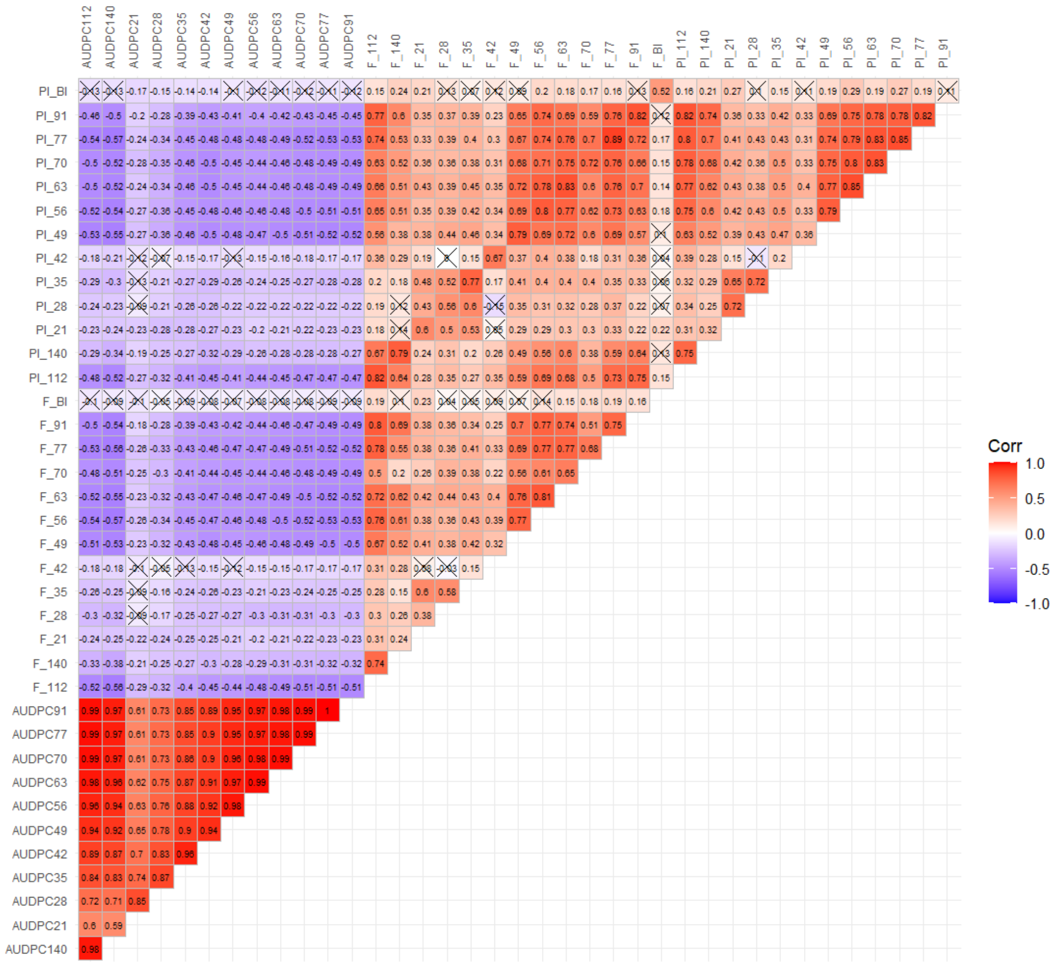

Supplementary Figure 2 - Heatmap representing pairwise Spearman's correlation coefficients between photosynthetic index of both photosystems I and II (PIabs representede by PI) and maximal efficiency of Photosystem II (Fv/Fm represented by F) measured in a sample of 215 *Pinus pinaster* plants inoculated with pine wood nematode at 21, 28, 35, 42, 49, 56, 63, 70, 77, 91, 112, and 140 days pos inoculation (dpi)**.** The correlation coefficients with a cross on top mean no significant correlation at a 5% significance level. Positive correlations are represented by reddish colours and negative correlations by bluish colours.

Supplementary Table 2. Results from fitting the linear mixed model to diameter and height data: overall phenotypic mean; *F*-test for the fixed effects of the family factor and the corresponding *p*-values; block variance estimate ($\hat{\sigma}^2_{Block}$) and family×block interaction variance estimate ($\hat{\sigma}^2_{Fam\times Block}$), along with the corresponding *p*-values from the tests of these variance components.

Traits	Phenotypic Overall mean	F value Family effects (p-value)	$\hat{\sigma}^2_{Block}$ (p-value)	$\hat{\sigma}^2_{Fam\times Block}$ (p-value)
Height (cm)	42.01	1.95 (0.1263)	46.31 (<0.001)	42.60 (<0.001)
Diameter (mm)	5.60	4.65 (0.005)	0.28 (0.008)	0.52 (<0.001)

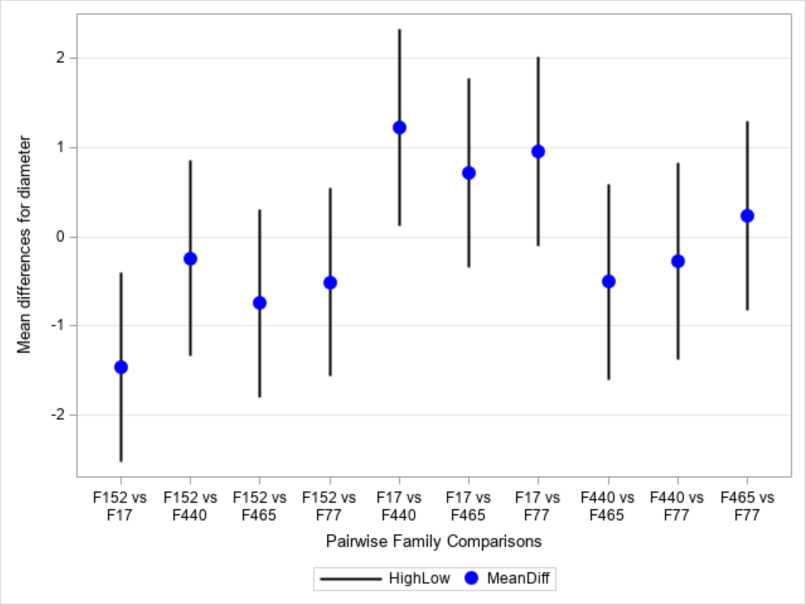

Supplementary Figure 3 - Tukey–Kramer 95% confidence intervals for all pairwise comparisons of the mean diameters (mm) of the five families F17, F77, F152, F440, and F465 (predicted means: F17 = 6.39; F465 = 5.67; F77 = 5.44; F440 = 5.17; F152 = 4.93).

Supplementary Table 3. Results for the empirical best linear unbiased predictors (EBLUP) of the block effect for plant diameter at the base (EBLUP-d) and total height (EBLUP-h)

Block	EBLUP-d	EBLUP-h
B1	0.4165	9.1939
B2	0.1535	0.7841
B3	-0.3746	-6.5284
B4	0.8708	9.1116
B5	-0.0508	0.9090
B6	-0.4733	-6.5237
B7	0.1329	2.7504
B8	-0.4339	-4.6099
B9	-0.2411	-5.0871

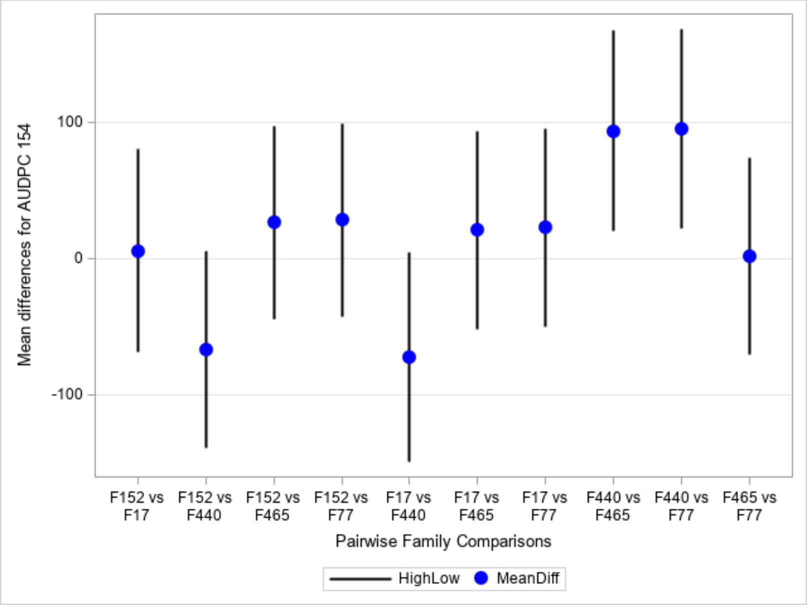

Supplementary Figure 4 - Tukey–Kramer 95% confidence intervals for all pairwise comparisons of the mean area under the disease progress curve (AUDPC) calculated at 154 days post inoculation (DPI) for the five families F17, F77, F152, F440, and F465 (predicted means: F440 = 301.45; F152 = 234.08; F17 = 228.66; F465 = 207.95; F77 = 206.07).

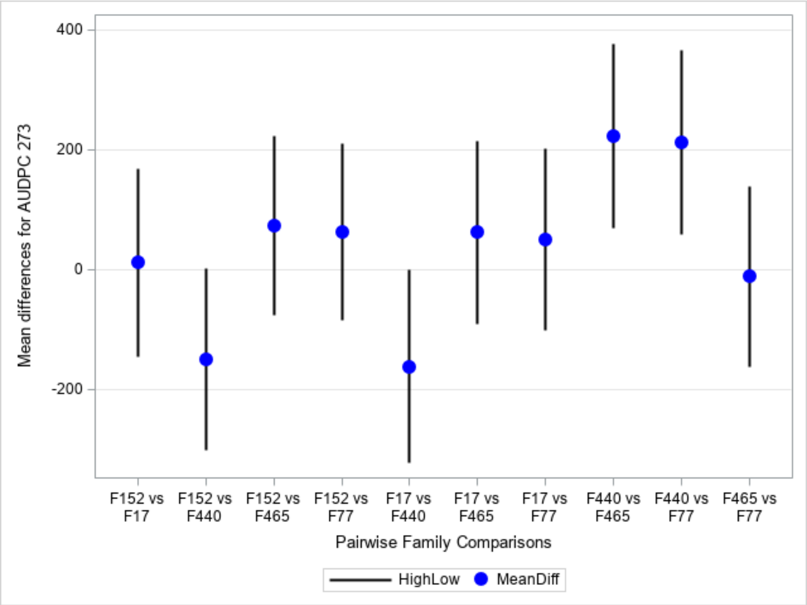

Supplementary Figure 5 - Tukey–Kramer 95% confidence intervals for all pairwise comparisons of the mean area under the disease progress curve (AUDPC) calculated at 273 days post inoculation (DPI) for the five families F17, F77, F152, F440, and F465 (predicted means: F440 = 624.22; F152 = 474.46; F17 = 462.87; F77 = 411.98; F465 = 400.76).

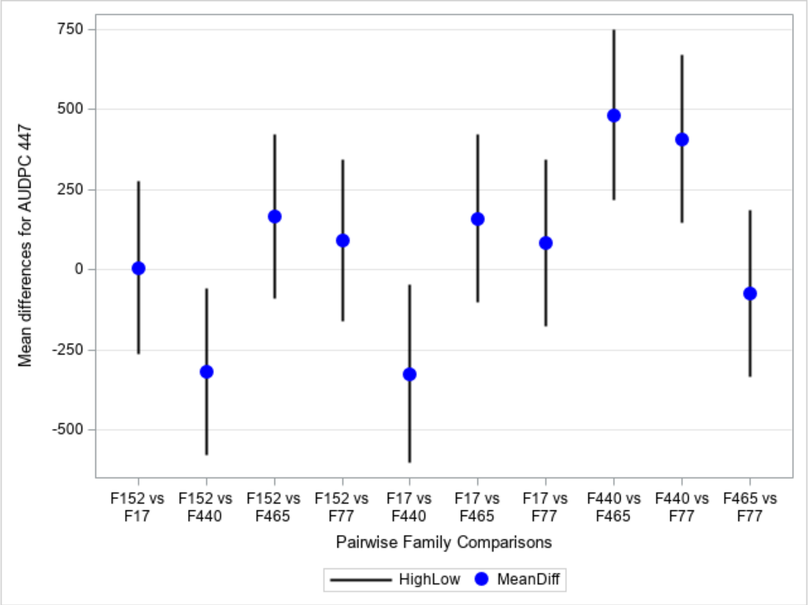

Supplementary Figure 6 - Tukey–Kramer 95% confidence intervals for all pairwise comparisons of the mean area under the disease progress curve (AUDPC) calculated at 447 days post inoculation (DPI) for the five families F17, F77, F152, F440, and F465 (predicted means: F440 = 1244.70; F152 = 926.93; F17 = 920.16; F77 = 835.98; F465 = 760.99).

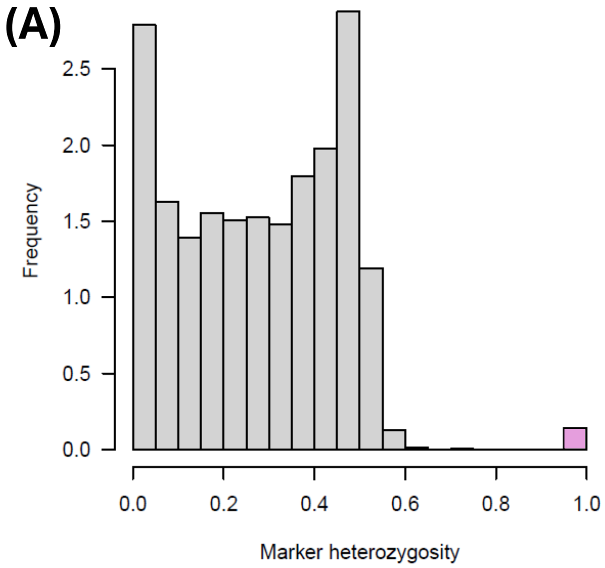

Supplementary Figure 7 - Marker density in the *P. pinaster* 510-plant panel. (A) Histogram plot of marker heterozygosity before SNP filtering. (B) Histogram plot of minor allele frequency (MAF) before SNP filtering. The pink bars are the SNPs that have been removed after filtering for heterozygosity > 0.8 and MAF ≤ 0.05.

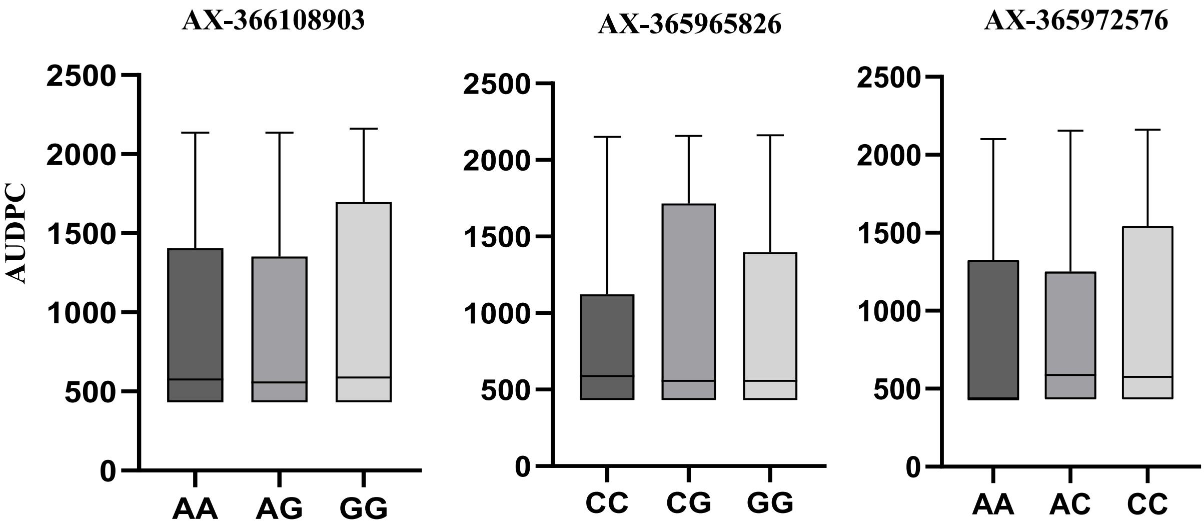

Supplementary Figure 8 - Boxplots of significant SNPs in the genome-wide association analysis, where the effect of alleles on the area under the disease progress curve (AUDPC) can be observed. The allele effects and inheritance mode could not be estimated as no differences were found in the single-marker ANOVA (p>0.05).

Supplementary Table 5. Expression results in Modesto *et. al* (2021) of the transcripts where SNPs with significant associations with AUDPC were located. The Log2(fold change) values were obtained by comparing susceptible plants (Sus) with controls (Cont) or resistant plants (Res) with controls (Cont) at 72 hours post-inoculation. Only significant results (FDR adjusted p-value ≤ 0.05) are represented.

**SNP marker**	**Transcript**	**Annotation**	**Log2(fold change)**	
			**Sus vs Cont**	**Res vs Cont**
AX-366080829	unigene7844	*DHQS*	0,32	0,38
AX-366099317	isotig28482	*CTPA1*	-0,57	-0,55
AX-366108903	unigene209735	*RPS26*	-	-
AX-366109081	NA	NA	-	-
AX-365965826	unigene25453	*OEE2*	0,93	0,75
AX-366030902	isotig41880	*PIP5K9*	-	-
AX-365972576	unigene1966	*EDS1L*	0,21	0,03

Supplementary Table 6. Epistatic effects among the six selected SNPs. Pairwise interactions were tested using separate mixed linear models in *ASReml*, with the corresponding SNPs and their interaction as fixed effects. The significance of each interaction was evaluated using Wald tests, and p-values were adjusted for multiple testing using the Bonferroni correction (α = 0.05/15 pairwise comparisons).

**Pairwise interactions**	**p-value**
AX-366099317:AX-366108903	0.2522
AX-366099317:AX-366109081	0.0542
AX-366099317:AX-365965826	0.1860
AX-366099317:AX-366030902	0.2724
AX-366099317:AX-365972576	0.7254
AX-366108903:AX-366109081	0.4620
AX-366108903:AX-365965826	0.5081
AX-366108903:AX-366030902	0.7523
AX-366108903:AX-365972576	0.1091
AX-366109081:AX-365965826	0.9472
AX-366109081:AX-366030902	0.6040
**AX-366109081:AX-365972576**	**0.0016**
AX-365965826:AX-366030902	0.5609
AX-365965826:AX-365972576	0.1155
AX-366030902:AX-365972576	0.0564

Supplementary Table 7. Effects of the significant pairwise interaction between AX-366109081 and AX-365972576 marker. 0 - homozygous for the reference allele; 1 - heterozygous; and 2 - homozygous for the alternative allele.

	Effect
AX-366109081_0:AX-365972576_0	0
AX-366109081_0:AX-365972576_1	0
AX-366109081_0:AX-365972576_2	0
AX-366109081_1:AX-365972576_0	0
AX-366109081_1:AX-365972576_1	**-699.32**
AX-366109081_1:AX-365972576_2	0
AX-366109081_2:AX-365972576_0	0
AX-366109081_2:AX-365972576_1	0
AX-366109081_2:AX-365972576_2	0
AX-365972576_0	0
AX-365972576_1	82.35367
AX-365972576_2	-47.3716
AX-366109081_0	0
AX-366109081_1	458.7009
AX-366109081_2	964.2951
(Intercept)	874.5382

Supplementary Table 8. Predicted mean values for the area under the disease progress curve (AUDPC), obtained by fitting a linear mixed model including the fixed effects of the two SNPs (AX-366109081 and AX-365972576) and their interaction. The '-' refers to non-existing allelic combinations in the half-sib families under study.

		**AX-365972576**		
		**AA**	**AC**	**CC**
**AX-366109081**	**TT**	874.54	956.89367	827.16844
	**TC**	1333.2409	**716.2745**	-
	**CC**	1838.83511	-	-

Supplementary Table 9. Statistical linkage disequilibrium among the six significant SNPs assessed using genotype-based r^2 estimates

	**AX-366099317**	**AX-366108903**	**AX-366109081**	**AX-365965826**	**AX-366030902**	**AX-365972576**
**AX-366099317**	1	0.014758848	0.00882801	0.014302029	0.01094599	0.00875531
**AX-366108903**	0.01475885	1	0.01952152	0.019790628	0.007217536	0.018656151
**AX-366109081**	0.00882801	0.019521518	1	0.002117212	7.74104E-05	0.00735948
**AX-365965826**	0.01430203	0.019790628	0.002117212	1	0.03276311	0.024981281
**AX-366030902**	0.01094599	0.007217536	7.74104E-05	0.032763114	1	0.003500394
**AX-365972576**	0.00875531	0.018656151	0.00735948	0.024981281	0.003500394	1
